# A Review of the Potential for Cardiometabolic Dysfunction in Youth with Spina Bifida and the Role for Physical Activity and Structured Exercise

**DOI:** 10.1155/2012/541363

**Published:** 2012-06-14

**Authors:** Kevin R. Short, Dominic Frimberger

**Affiliations:** ^1^Section of Diabetes & Endocrinology, Department of Pediatrics, University of Oklahoma Health Sciences Center, Oklahoma City, OK 73104, USA; ^2^Section of Pediatric Urology, Department of Urology, University of Oklahoma Health Sciences Center, Oklahoma City, OK 73104, USA

## Abstract

Children and adolescents who have decreased mobility due to spina bifida may be at increased risk for the components of metabolic syndrome, including abdominal obesity, insulin resistance, and dyslipidemia due to low physical activity. Like their nondisabled peers, adolescents with spina bifida that develop metabolic risk factors early in life have set the stage for adult disease. Exercise interventions can improve metabolic dysfunction in nondisabled youth, but the types of exercise programs that are most effective and the mechanisms involved are not known. This is especially true in adolescents with spina bifida, who have impaired mobility and physical function and with whom there have been few well-controlled studies. This paper highlights the current lack of knowledge about the role of physical activity and the need to develop exercise strategies targeting the reduction of cardiometabolic risk and improving quality of life in youth with spina bifida.

## 1. Introduction

Spina bifida (SB) is a congenital abnormality characterized by the incomplete closure of the spinal column. The majority (>90%) of cases are classified as meningomyelocele, in which the spinal cord protrudes through the spinal column, resulting in nerve damage and physical disabilities including lower limb paralysis and disrupted bladder or bowel function. Widespread public advertising and clinical advocacy campaigns have been used to promote the intake of adequate dietary folic acid during pregnancy, since this strategy has been shown to reduce the risk of developing SB. Nevertheless, the current prevalence of SB in the United States is estimated to be close to 1 in 1,000 pregnancies [1]. With better surgical treatments and early medical care infant survival rates rose from 83% in 1979–83 to 91% in 1989–94 [2]. A larger national survey covering 1995–2001 reported infant survival rates of 92% [3]. Thus, in the United States ~25,000 children ages 0–19 years and ~166,000 total people are currently affected by SB [4, 5].

As a result of improved surgical treatments and early medical care, children with SB can expect longer lives today than in the past [2]. In recent decades spinal repair and shunting techniques have improved, complications have declined, and survival rates have increased [1, 2, 6, 7]. Although life span for people with SB has increased, there remain physical limitations and mobility issues that require attention. Additionally, a new health challenge that must be considered is the potential for cardiometabolic disease risk, which may result from the physical deconditioning that occurs in people with disabilities [8]. As described by Rimmer et al. [8] in their recent review, people with physical disabilities spend less time performing physical activities than their nondisabled peers. The consequences of a sedentary lifestyle in all people include physical deconditioning and increased risk of developing obesity, insulin resistance, and cardiovascular disease. People with disabilities such as SB or spinal cord injury (SCI), especially those confined to wheelchairs, are prone to develop what Rimmer et al. [8] termed “disability-associated low energy expenditure deconditioning syndrome,” in which sedentary lifestyle creates a vicious cycle of further deconditioning, disability, and disease risk. This cyclic problem worsens with advancing age, but strategies such as exercise programs tailored to meet the specific needs of the individual can be an effective approach to attenuate or reverse the progression of functional disability and disease risk. To date, the presence of cardiometabolic disease risk in people with SB is not well described. Additionally, there have been only a few published studies that reported the results of exercise training programs for children with SB. Those studies, however, have been small and primarily focused on physical function rather than cardiometabolic health. In fact, a recent systematic review concluded that most of the publications on exercise programs for SB were not of high quality due to small sample size and poorly defined outcomes [9].

The purpose of this narrative review is to describe the current scientific literature on cardiometabolic risk in youth with SB and the potential for structured exercise intervention programs to improve health and function in that population. The volume of existing literature in this area is relatively small and therefore neither a structured systematic review of the evidence, nor a meta-analysis of the results of multiple exercise studies was feasible. Thus, our goal is to outline what is known about the potential cardiometabolic consequences of SB, to highlight both the strengths and weaknesses of selected studies, to identify gaps in knowledge, and to suggest future directions that would produce the type of evidence that will ultimately guide improved clinical care and lifestyle recommendations for people with SB. Our review of the literature covers primarily papers published with in the past 25 years that could be identified through PubMed and cross-referencing citations of published papers. We included some studies with small sample sizes because so little has been published to date, but excluded individual case studies. Although the focus of this narrative review is on SB, we have included selected results obtained from studies of SCI and childhood obesity because of their relevance to understanding the potential consequences of physical disability, deconditioning, and cardiometabolic risk in youth with SB. 

## 2. Body Composition, Physical Function, and Cardiometabolic Risk

### 2.1. Health and Function in Children with SB

Although the prevalence of diabetes and cardiovascular disease in the SB population is not known, children and adults with SB may be at increased risk for metabolic and vascular dysfunction because of their body composition, physical function, and clinical blood test results (Table 1). One of the most common findings has been that body mass index (BMI) and percent body fat are increased in people with SB relative to people without SB [10–16]. Although it has been reported that overweight and obesity prevalence are increased in both children and adults with SB [10–14, 16], Dosa et al. [15] found in their study of 203 people that obesity prevalence, based on BMI, for children 6–19 years old with SB (8–18%) was similar to current general population values for children in the United States. However, they found that for adults >20 years old obesity rates in people with SB (37%) were higher than in the general population [15]. The sample size in that study was larger than most others in the SB literature, but it is unclear if the results are generalizable since, to our knowledge, there are no large-scale surveys of obesity prevalence in people with SB. It is important to note, though, that classifying overweight and obesity in people with SB according to BMI standards, as it is done in the nondisabled population, may underestimate the true extent of obesity in people with SB. Nelson et al. [12] proposed defining obesity in children affected by SB or SCI as the presence of abdominal fat measured by dual X-ray absorptiometry that exceeds 30% of tissue mass in boys or 35% in girls, respectively. Using this approach they reported that 18 out of 34 (~53%) children with SB tested in their center met the criterion for obesity based on standardized BMI *z*-score, but that 25 out of 34 (~74%) would be classified as obese based on abdominal fat content. Those results underscore the fact that BMI does not provide information about the composition of body mass and can often mask offsetting changes in the proportions of fat and lean tissue that occur over time within individuals, or differences that exist between comparison groups. Furthermore, BMI standards developed for nondisabled children have not been validated for children with SB. To do so would require careful control for the level of ambulation and spinal cord involvement and might prove difficult because of the wide range of physical abilities and spinal cord deficits in people with SB. Adopting a classification system for overweight or obesity based on body composition, however, is also challenging in clinical settings since there are several potential methods (e.g., skinfolds, air displacement plethysmography, underwater weighing, bioelectrical impedance, dual energy X-ray absorptiometry, and magnetic resonance imaging) that require expertise and resources that may not be available in some centers. Nevertheless, in research settings, assessing body composition, particularly abdominal fat, should be included in future assessments of metabolic risk in youth with SB.

In addition to increased body fatness, children and young adults with SB typically are reported to have reduced aerobic fitness and muscular strength [10, 11, 17]. These deficits in physical capacity are reported in both ambulatory individuals tested using leg cycling or on a treadmill, and in nonambulatory individuals tested using arm crank ergometry, respectively. The study of Widman et al. [10] was notable because there were 37 nonambulatory adolescents with SB who completed assessments of upper body muscle strength and maximal aerobic capacity testing, and the results were compared to 34 age-matched control adolescents without SB who completed the same tests in the same research center. In two other studies [11, 17], measurements of muscle strength, and aerobic capacity during leg cycling were compared against previously published data from nondisabled people and found to be up to 30–40% lower in people with SB. These differences are large enough to support that physical function deficits are present in people with SB. However, reliability and normative values for body composition and physical function can vary among laboratories due to differences in test equipment, test operators, and regional population characteristics. Thus, research studies on SB should ideally include comparison data from age- and sex-matched controls studied in the same laboratory. Comparison values for aerobic capacity during arm crank ergometry were not provided in two of the studies cited previously [11, 17]. This is a limitation since the number of nonambulatory people with SB is substantial; for example, ~90% of SB patients in our clinical center rely on wheelchairs and are nonambulatory. Thus, further descriptive data are needed to fully characterize the upper body exercise capacity of youth with SB.

As a result of low aerobic capacity and muscle strength, activities of daily living may be relatively more difficult for people with SB. Bruinings et al. [18] reported that the oxygen cost (work economy) of walking or wheelchair use at fixed speeds for children and young adults with SB was similar to, or even less than that of age-matched people without SB. However, for ambulatory people with SB, self-selected walking speed was 14% slower compared to nondisabled controls [18]. More importantly, because of their reduced peak aerobic capacity, activities of daily living were relatively more demanding since they required a higher portion of aerobic reserve capacity for both ambulatory and nonambulatory people with SB. Thus, it is not surprising that the amount of daily physical activity performed by children and adults with SB is lower than nondisabled comparison groups [11, 19, 20]. Likewise, the reported quality of life is lower in people with SB, particularly in those who are nonambulatory [21–24]. In the study by Abresch et al. [21], quality of life was similarly reduced in children with either SB or SCI compared to healthy controls. Interestingly, though, the presence of obesity adversely affected quality of life scores in healthy control children, it had no further effect on the scores of children with SB or SCI in that study. It remains to be shown whether quality-of-life assessments are improved following physical activity interventions.

Given the increased presence of obesity and decreased physical activity and physical function, it is plausible to expect that the components of metabolic risk would be adversely affected by the presence of SB. There are, however, only a few studies that have measured the components of metabolic syndrome or associated risk factors in children or adults with SB. The available reports showed that fasting glucose, insulin, cholesterol, triglycerides, and blood pressure were, on average, not different in people with SB compared to either non-SB control groups or published normal values (Table 1) [12, 25, 26]. Some studies have reported values for clinical blood tests but did not include group means and control values, so the effect of SB was difficult to determine [27]. Exceptions to those trends were reported by Rendeli and colleagues, who found that total- and VLDL-cholesterol were increased in girls with SB, though not in boys with SB [26], and that circulating homocysteine was increased in boys and girls with SB [28]. The difference in total cholesterol outcomes between boys and girls in the former study [26] appeared to be attributable to differences in walking ability. Total- and VLDL-cholesterol values were higher in girls who did not walk compared to those who walked independently or with assistance, but this effect of walking ability was not apparent in boys. This observation may be related to the finding that girls are, in general, less likely to be physically active than boys during childhood and adolescence [29]. It should also be noted, however, that the data in the study by Rendeli et al. [26] were not corrected for potential differences in age or maturation between sexes and therefore require confirmation.

Although the group means reported in most of the studies cited above revealed little or no differences between people with and without SB, the results require confirmation in larger studies, and there is still reason for concern. For example, assessment of vascular function in a small group of men with SB demonstrated that arterial diameters were reduced and sheer stress on the vascular wall was increased compared to nondisabled controls, a result that is predictive of endothelial dysfunction [30]. Additionally, in a Dutch cohort of 31 adolescents and young adults with SB, ~90% had one or more risk factors for cardiovascular disease based on the Framingham Heart Study criteria, which includes assessments of total- and HDL-cholesterol, systolic blood pressure, and smoking [27]. Similarly, Nelson et al. [12] reported that fasting insulin resistance (measured using the calculation of homeostatic model of assessment for insulin resistance, HOMA-IR) was significantly increased in children and young adults with SB or SCI who were obese. Furthermore, 70%–75% of study participants with SB and 80–100% of participants with SCI had low HDL-cholesterol, independent of the presence of obesity. Metabolic syndrome (defined as the presence of three or more risk factors from a panel including obesity assessed by BMI, percent body fat or waist circumference; insulin resistance assessed by fasting glucose, fasting insulin, or impaired glucose tolerance; hypertriglyceridemia; low HDL-cholesterol; hypertension) was present in 32% of people with SB and 50% of people with SCI, compared to 20% of nondisabled controls [12]. The presence of those adverse clinical outcomes in the SB and SCI groups could be predominantly ascribed to the copresence of obesity, just as it was in the nondisabled control group. However, because youth with SB (and SCI) had higher rates of metabolic syndrome, which represents the clustering of adverse clinical conditions, and because the prevalence of obesity is elevated in the SB population, the results of that study [12] and the work of Buffart et al. [27] suggest that clinicians should carefully monitor young people with SB for these signs of disease risk.

### 2.2. Additional Insights into Health and Function from Studies of SCI

Although the etiology of their disabilities is clearly different from SB, people with limited use of their legs due to spinal cord injury (SCI) and who rely on wheelchairs for mobility face many of the same health concerns as people with SB. In the United States, there are >1.2 million people with spinal cord injury (SCI) and ~1,500–2,000 new pediatric cases of SCI per year [31]. Because of similar physical challenges and sedentary lifestyle, individuals with SCI, like those with SB, face an increased risk of cardiometabolic disease. As with SB, children with SCI are likely to have obesity characterized by increased abdominal fat and reduced lean mass [16, 32]. In the study by Liusuwan et al. [16] the magnitude of increase in body fat and decrease in lean body mass relative to normal weight or overweight nondisabled control groups was greater in youth with SB than with SCI. This finding suggests that the long-term health consequences of obesity for SB may be more pronounced than for SCI.

Although there are relatively few studies of metabolic risk factors in adults with SB, there are relevant data in the SCI population that could be used to predict longer-term health risks in the SB population. For example, a recent analysis of 134 Swedish adults with SCI revealed that one-third of people met the criteria for cardiovascular risk intervention based on the Framingham Risk Equation and the Systemic Coronary Risk Evaluation models, which include measures of cholesterol, blood pressure smoking, age, and sex [34]. When the authors of that study included BMI >22 kg/m^2^ as a potential measure of overweight deemed appropriate for people with SCI, nearly 80% of the group reached the higher risk classification worthy of clinical intervention [34]. Additionally, as shown in children and young adults with SB, HDL-cholesterol has been reported to be reduced in people with SCI [35]. Additional markers of cardiometabolic disease risk that are reported to be increased in people with SCI include the inflammatory proteins, C-reactive protein, and interleukin-6 [35, 36], neither of which have been reported in people with SB to our knowledge. Furthermore, Nash et al. [37] made the observation in young adults with SCI that fasting triglycerides were normal, but the elevation in triglycerides was ~35% higher following a high-fat meal compared to recreationally active nondisabled men. They proposed that the meal stimulus revealed a type of dyslipidemia that is undetected when only fasting measurements are used. This situation is analogous to carbohydrate metabolism, whereby fasting glucose may be in the normal range but postprandial blood glucose is elevated, revealing impaired glucose tolerance. The finding by Nash et al. [37], however, was based on data on only three young men with SCI and therefore needs to be replicated in both the SCI and SB populations. Postprandial hyperlipidemia could be an important clinical outcome as it has been shown to be increased in nondisabled people with hypertension and cardiovascular disease [38] and may therefore represent an early marker for disease risk applicable to adolescents with SB. 

### 2.3. Health Consequences of Obesity and/or Sedentary Lifestyle in Nondisabled Youth

The potential metabolic risk in children with SB resembles in many ways the current problem of obesity and sedentary lifestyle in all adolescents. According to body mass index criteria for obesity (≥the 95th percentile for age and sex) ~18% of children in the United States are obese [39]. While overnutrition is a key contributor to the development of childhood obesity, there is also a role for physical activity, and half of American children fail to reach the recommended 60 minutes per day of moderate-to-vigorous physical activity [29, 40]. Abdominal adiposity in children is a strong predictor of insulin resistance and is associated with features of metabolic syndrome such as dyslipidemia, hypertension, and increased circulating inflammatory markers, all of which have been more widely studied in nondisabled children than children with SB [41–46]. It is estimated that 25–50% of overweight adolescents meet the criteria for metabolic syndrome [47]. More importantly, longitudinal studies show that obesity and dyslipidemia that are present during childhood often persist into adulthood and predict the development of cardiovascular disease [48–50], which makes obesity and sedentary lifestyle in today's youth such a widespread concern. Thus, lifestyle or other clinical strategies need to be developed to address these concerns for youth in general and tailored to meet the special needs of children with SB. 

## 3. Exercise as a Means of Lowering Metabolic Risk

### 3.1. Exercise Programs for People without SB

It is well established that exercise, alone or in combination with dietary strategies aimed to reduce body weight, can be effective for the prevention of diabetes and metabolic syndrome in nondisabled children and adults [51–55]. Extensive literature has also shown that exercise performed at least 2-3 days per week can improve insulin sensitivity, reduce abdominal fat, and increase HDL-cholesterol, in addition to providing numerous other health benefits in nondisabled children and adults [56–63]. Similarly, when exercise is performed up to 15 hours before a meal the circulating postprandial triglyceride concentration is reduced, a result that is attributed to activation of lipoprotein lipase by muscle contraction [64–67]. Unfortunately, in the United States, recent observational studies have reported that less than half of children and adults meet the current recommendations of 300 and 150 minutes per week, respectively, of moderate-to-vigorous physical activity [29, 40, 68]. Likewise, due to the physical limitations described a common clinical observation is that many people with SB have low physical activity. The extent of this perceived problem is not yet quantified, since descriptive data for the participation rates in physical activity, including structured exercise or sports or activities of daily living, are to our knowledge not available for the SB population.

### 3.2. Exercise for Children with SB

Recent work suggests that some adolescents with SB can successfully participate in sports and should be encouraged to do so for psychosocial reasons [69, 70]. Buffart et al. [22] reported that children and young adults with SB had lower reported quality of life scores than a nondisabled comparison group, and ~60% had difficulty with activities of daily living. However, after controlling for ambulation status and sex (walkers and males reported fewer difficulties, resp.), daily physical activity and aerobic fitness were positively correlated with quality of life and inversely correlated with physical difficulties. These results support the possibility that appropriately tailored physical activity programs could improve perceived quality of life for young people who currently have low habitual activity. However, the most feasible and effective types of exercise for people with SB are not well defined.

To date, physical therapy strategies employed with people affected by SB have focused on muscle strengthening exercises and orthopedic supports and assistive devices meant to aid ambulation and postural control [71–73]. Similarly, the few exercise intervention trials performed with people with SB-targeted improvements in muscle size, muscle function, or aerobic capacity since these outcomes are likely to translate to better mobility and performance of activities of daily living [74–77]. However, most of those studies used small sample size and required replication. 

The first study to our knowledge to report the feasibility and effectiveness of exercise in SB was published by Ekblom and Lundberg in 1968 [74]. In that study, seven adolescents with SB, seven with cerebral palsy, and three with other forms of spinal paraplegia, completed a low volume (30 minutes twice per week for 6 weeks) of moderate-intensity wheelchair exercise consisting of wheelchair driving and upper body strengthening activities. This program resulted in improvements in upper body fitness in the SB group as shown by a 10-11% reduction in oxygen uptake and heart rate and a 40% reduction in blood lactate during submaximal arm crank exercise. During maximal arm crank exercise, work output was 21% higher after training although peak oxygen uptake did not change; suggesting that both submaximal and maximal work economy was increased. Since that report, however, there have been only two investigations in which nonambulatory children or adults with SB performed upper body exercise training and fitness outcomes were reported. Andrade et al. [75] had eight children with SB, five who were ambulatory, and three who wheelchair users, complete a once-weekly supervised program of upper body aerobic and resistance exercise, which resulted in improvements in upper body strength after 10 weeks. Similarly, O'Connell and Barnhart [76] enrolled three children with SB and three with cerebral palsy, all of whom were wheelchair users, in a program of upper body resistance training performed three times per week for nine weeks. Despite a wide range of ages (5–16 years old) and abilities of the participants, the exercise program resulted in an increase in upper body strength (70–200% improvement) and self-propelled wheelchair distance covered in 12 minutes (29%). Although each of those studies demonstrate the potential benefits of upper body endurance and strengthening exercises on physical function for children with SB, the results are difficult to generalize due to the small sample sizes and heterogeneity of the participants. None of the studies assessed the impact of the exercise program on body composition or cardiometabolic risk factors such as lipids, glucose, insulin, blood pressure, or vascular function. Additionally, the pretraining familiarization and testing protocols were not well described so it is possible that at least part of the improvements ascribed to exercise training may be due to learning effects that can occur when novice participants gain increased awareness to study goals and are more familiar with the testing environment at the posttest compared to the baseline measurement.

More recently, de Groot et al. [77] conducted a home-based walking trial for ambulatory children with SB. Children in the intervention group were provided with a treadmill and instructed to exercise for up to 30 minutes per session in two days per week for 12 weeks in addition to their normal daily activities, while children in the standard-of-care control group maintained their habitual lifestyle patterns. At the end of the program, the walking group increased the distance they could walk during a 6-minute test by 38%, and this improvement was maintained for at least three months, whereas the walking distance in the control group was unchanged. Similarly, peak aerobic capacity and walking economy improved in the walking group but not the control group. This study demonstrates that children with SB can improve their functional ability by performing a structured, progressive intensity exercise program. It remains to be shown, however, if these positive results can be extended to people with SB who are nonambulatory, and whether the benefits of the exercise translate to improvement in metabolic and vascular outcomes.

### 3.3. Exercise Programs for People with SCI

The value of considering exercise intervention programs for people with SCI is that there have been far more studies conducted compared to the situation for SB [78, 79]. Supporting the small evidence base for SB, the more extensive literature on SCI indicates that regular exercise is beneficial for people with limited mobility, which has led to the development of structured exercise recommendations for spinal cord injured people [80–82]. In adults with SCI cross-sectional studies demonstrate that the volume of daily physical activity is inversely associated with obesity and several metabolic risk factors, and positively related to aerobic fitness and quality-of-life assessments [83–85]. Likewise, beneficial changes in health and functional outcomes are observed when adults with SCI complete exercise training or functional electrical stimulation protocols [35, 79, 86–90]. Some of these studies used small sample sizes [87, 90, 91], but despite that limitation, the potential for metabolic benefits is promising. For example, de Groot et al. [87] showed that after 8 weeks of upper body exercise, men with SCI who performed high-intensity interval training had better improvement in aerobic capacity and fasting lipid profile than those who performed continuous moderate-intensity exercise. Although that study only included 3 men per group and requires confirmation, the results are consistent with several recent reports demonstrating that, compared to moderate-intensity exercise, aerobic interval training produced equal or greater fitness and metabolic benefits for obese adolescents and adults with heart disease or metabolic syndrome [92–96]. In the home-based walking program for children with SB described previously, de Groot et al. [77] used a modified version of this type of aerobic interval exercise. Thus, using short segments of higher intensity exercise could be an effective, time-efficient means to promote favorable fitness and metabolic adaptations in youth with SB. This and other exercise strategies need to be tested systematically, with the types of adaptations required to accommodate the needs and abilities of the individual carefully documented and disseminated.

## 4. Summary and Recommendations

In summary, although it may seem intuitive that exercise should be a cornerstone of healthy lifestyle recommendations for SB patients, there are so few studies on the effects of exercise in this population that it is unclear whether specific types of activity are more effective than others. Recent exercise recommendations have been developed for adults with SCI and consist of at least 20 min of moderate-to-vigorous intensity aerobic activity performed two times per week, and strength training exercises performed two times per week, comprised of three sets of 8–10 repetitions of exercises targeting each major muscle group [80]. Although it is plausible that this recommendation is appropriate and effective for children with SB, it is still unclear whether adjustments are needed to address the physical challenges that accompany SB or to meet specific health and function needs. For youth with SB who have limited use of their lower limbs, exercise must necessarily rely on the upper body and trunk muscles. For wheelchair users, this often means that there are fewer opportunities for physical activity and sports. Children with SB face barriers of access to appropriate exercise facilities and must rely on adults to help organize and supervise sports or physical play opportunities, which may be lacking in many communities. Thus, there is a potential role for healthcare providers to work with local fitness centers and schools to develop activity programs that incorporate structural exercise, sports programs like wheelchair basketball, and age- and function-appropriate movement games. Since upper body exercise activates less muscle mass than typical leg exercise it may be necessary to carefully regulate intensity or duration of activity, or to incorporate electrical stimulation of the lower limb musculature to ultimately reduce body fatness, insulin resistance, inflammation, and/or hyperlipidemia. Our premise is that distinct exercise approaches that vary in the volume, intensity, frequency, and/or mode of activity could be developed to target different outcomes in physical function and metabolic health. Achieving that goal will require detailed investigations that address the feasibility and effectiveness of exercise for children with SB. In the meantime, clinicians that work with patients with SB should encourage them to strive to increase their habitual physical activity on most or all days of the week, since the literature supports that this may help, improve physical function and perceived quality of life.

## Figures and Tables

**Table 1:** Summary of published outcomes for body composition, physical function, and metabolic and vascular risk factors in people with spina bifida (SB).

Outcome	Increased in SB	Decreased in SB	Not different in SB
Body mass index, for age and sex	- Non-amb children [10]		- Amb and non-amb children [15]
	- Amb children [11]^∗^		
	- Children and young adults [12]		
	- Amb and non-amb adults [15]		
	- Children [16]		

Total body fat (%)	- Non-amb children [10]		
	- Children and young adults [12]		- Amb children [11]^∗^
	- Children [13, 14, 16]		

Abdominal fat (%)	- Children and young adults [12]		
	- Obese children [25]^∗^		

Lean body mass (%)		- Children [14, 16]	

Aerobic capacity		- Non-amb children [10]	
		- Amb children [11]^∗^	
		- Non-amb children and young adults [17]^∗^	
		- Amb children and young adults [17]^∗^	
		- Amb children [33]	

Muscle strength	- Non-amb children [10]	
	- Amb children [11]^∗^	
	- Non-amb children and young adults [17]^∗^	
	- Amb children and young adults [17]^∗^	

Absolute energy cost of walking or wheelchair use		- Non-amb children and young adults [18]	- Amb children and young adults [18]

Requirement of physical reserve for activities of daily living	- Non-amb children and young adults [18]		
	- Amb children and young adults [18]		

Total daily physical activity	- Amb children [11]^∗^	
	- Non-amb children [19]	
	- Amb children [19]	
	- Non-amb children and young adults [20]^∗^	
	- Amb children and young adults [20]^∗^	

Glucose		- Children and young adults [12]	- Obese children [25]^∗^

Insulin			- Children and young adults [12]

HOMA-IR	- Obese children [25]^∗^		- Children and young adults [12]

Total cholesterol	- Amb and non-amb girls [26]		- Children and young adults [12]
			- Amb and non-amb girls [26]

LDL-cholesterol			- Children and young adults [12]
			- Amb and non-amb girls [26]

HDL-Cholesterol			- Children and young adults [12]
			- Amb and non-amb girls [26]

Triglycerides			- Amb and non-amb children [26]

Homocysteine	- Amb and non-amb children [28]		

Blood pressure	- Amb and non-amb children and young adults [27]		- Obese children [25]^∗^

All comparisons refer to outcomes for people with SB relative to people without SB. Results from people with SCI or other spinal disorders are not included in this table according to the descriptions presented in the individual studies. ^∗^Comparison is relative to published or unpublished values from prior studies; all other investigations used a specifically recruited comparison group within their study design. Amb: ambulatory; Non-amb: nonambulatory. If not specified, ambulatory status was not stated in the cited study. In some studies “amb and non-amb” designation is used because the results for ambulatory and nonambulatory participants were not presented separately. The designation for children and adults in the cited studies is defined as <18 or ≥18 years old, respectively. In some studies, results from children and adults were not presented separately.
